# Leptin regulates Granzyme-A, PD-1 and CTLA-4 expression in T cell to control visceral leishmaniasis in BALB/c Mice

**DOI:** 10.1038/s41598-017-15288-7

**Published:** 2017-11-07

**Authors:** Alti Dayakar, Sambamurthy Chandrasekaran, Jalaja Veronica, Vadloori Bharadwaja, Radheshyam Maurya

**Affiliations:** Department of Animal Biology, University of Hyderabad, Hyderabad, 500 046 India

## Abstract

Visceral leishmaniasis (VL) is responsible for several deaths in malnourished children accompanied by diminished circulating leptin and impaired cell-mediated immunity. Typically, leptin deficiency is associated with the Th2 polarization that markedly coincides with the pathogenesis of VL. The aim of the present study was to unravel the prophylactic role of leptin in malnutrition-coupled VL mice. Interestingly, we observed that *L. donovani* infection itself reduces the serum leptin levels in malnutrition. Exogenous leptin restored severe body weight loss and parasite load in the spleen and liver of malnourished infected mice compared to controls. Leptin increases functional CD8+ T-cell population, Granzyme-A expression down-regulates anergic T-cell markers such as PD-1 and CTLA-4. It was also noticed that, leptin suppresses GM-CSF mRNA expression in parasite favored monocytes and reduced arginase activity in bone marrow derived macrophage indicate macrophages dependent T-cell activation and proliferation. Leptin-induced IFN-γ, IL-2, and TNF-α cytokines in the culture supernatant of splenocytes upon soluble leishmanial antigen (SLA) stimulation and significantly up-regulates serum IgG2a titers, which help to generate Th1 immune response in VL. Furthermore, leptin induced a granulomatous response and restored *L. donovani* induced tissue degeneration in the liver. Altogether, our findings suggest the exogenous leptin can restore T cell mediated immunity in malnourished VL mice.

## Introduction

Visceral Leishmaniasis (VL) is a vector borne infectious disease caused by the protozoan parasite *Leishmania donovani* in the Indian subcontinent. VL majorly affects the undernourished population especially children (5–14 years) in endemic regions of tropical and subtropical countries, and it is the most severe clinical form of the disease characterized by systemic infection to vital lymphoid organs such as lymph nodes, liver, spleen, and bone marrow^1^. The global burden of VL is about 400,000 new cases and >40,000 deaths per year^2^. The most affected countries are Sudan, Ethiopia, Brazil, and the Indian subcontinent, which is accounted for 90% of cases^3^.

Successful treatment of VL depends on the induction of cellular immunity together with the production of the proinflammatory cytokines owing to Th1 response primed mostly by interleukin IL-12 produced from dendritic cells and macrophages^4,5^. Production of IL-12 by antigen-presenting cells (APCs) and interferon IFN-γ by T-cells is crucial for controlling parasite growth by inducing nitric oxide (NO) signalling^6,7^. The host immune response was skewed towards IL-10, transforming growth factor (TGF)-β, or IL-4 producing Th2 cytokines and IL-10 producing T-regulatory cells, suppressing host immunity and help parasite survival^8,9^. However, IL-10 also protects the host from tissue damage caused by excessive inflammatory cytokines^10^. Exhaustion of CD8+ T-cells has been defined as antigen-specific effector T-cells dysfunction with sustained expression of inhibitory receptors including programmed death-1 (PD-1) and decreased effector cytokine production in chronic parasitic diseases as in toxoplasmosis and cutaneous leishmaniasis^11,12^. A chronic murine infection with arginase-deficient *L. major* demonstrated that impaired priming of T-cells can result in PD-1 overexpression, impairment of acquired immunity, and CD8+ T-cell exhaustion^13^. Although VL is asymptomatic, protein-energy deficiency increases the risk of rapid development of the symptomatic clinical disease.

Protein-energy deficiency is a major concern of malnutrition, affects 826 million people globally and accounts for 2.2 million annual deaths, of which 95.9% only in developing countries^14,15^. Malnutrition is associated with immune suppression thereby increasing the incidence of infections and mortality^16^, which affects both innate and acquired immunity^17^. It is associated with low circulating leptin levels^18^; thereby highly susceptible to infections due to defective cytokine production^19^. Leptin is a pleiotropic molecule produced by adipose tissue. It functions as a hormone as well as a cytokine, and its levels are always in proportion to the body fat mass^20^. It plays an important role in the regulation of immune response via T-lymphocytes proliferation^21^, thymic homeostasis, and activation of monocyte/macrophages and dendritic cells^22^. Leptin induces the phagocytic activity of macrophages and prevents the apoptosis of various immune cells involved in innate and adaptive immune response^23^. Leptin deficiency is associated with Th2 polarization characterized by increased production of IL-10, IL-4, and downregulation of Th1 response, which coincides with VL pathogenesis^24,25^.

Previous studies have been reported that the systemic circulating leptin deficiency in malnutrition is also correlated in several infectious diseases such as tuberculosis^26^, pneumonia^27^, sepsis^28^, colitis^29^, viral immunity^30^ amoebiasis^31^ and leishmaniasis^32,33^ due to defective cytokine production^19,25^. Leptin has been proved as an effective mucosal vaccine adjuvant against *Rhodococcus equi* ^34^ and *Helicobacter pylori* ^35^ and its proper signalling in gut mucosal epithelial cells offer more resistance against amoebiasis caused by *Entamoeba histolytica* ^36^. We have also demonstrated that leptin can augment host protective immune response during experimental VL^32,33^. Leptin induces the phagocytic activity of human macrophages against *L. donovani* infection by enhancing the phagolysosome formation and oxidative killing of the parasite via intracellular ROS generation^33^. Recently we demonstrated that recombinant leptin treatment reduced splenic parasite burden compared with non-treated infected mice fed with normal diet. Leptin also induces the innate immune response in bone marrow derived antigen-presenting cells, resultant an increase of nitric oxide and proinflammatory cytokines (IFNγ, IL-12, and IL1β) response in SLA stimulated splenocytes^37^. Moreover, exogenous leptin induced IFNγ production in both CD4+ and CD8+ T cells lymphocytes, indicating its ability to induced cell-mediated immunity in mice fed with normal diet^37^. To follow up on our earlier report and to test if leptin administration could be protective in malnutrition coupled VL^38^, we investigated the effects of exogenous leptin in malnutrition coupled *L. donovani* infection in BALB/c mice. The data presented here indicate that leptin supplementation may have positive effects in control of VL.

## Results

### Leptin regulated rapid body weight loss in BALB/c mice

**Figure 1:**
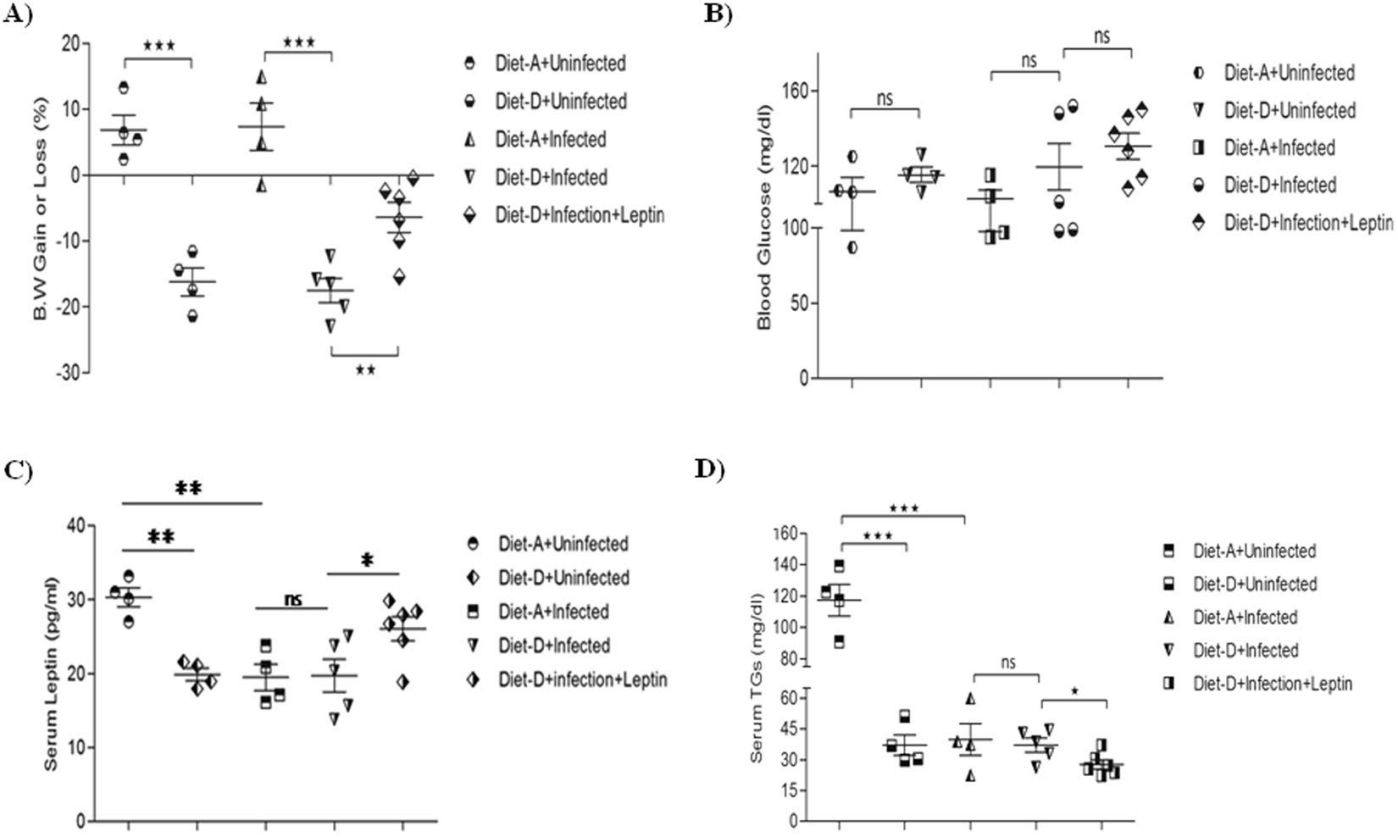
Assessment of body weight (B.W) and blood chemistry upon leptin treatment. (**A**) The body weight was significantly reduced in the diet-D infected/control groups compared to its control. However, leptin-treated diet D group shown rapid control in their body weight loss. (**B**) Post-prandial blood glucose (mg/dl) levels were unaltered all cases. (**C**) Serum leptin in uninfected groups of diet-D significantly reduced as compared to diet-A control groups and leptin treated diet-D groups significantly restored serum leptin level. Although we did not treated Diet-A groups in this study but we have observed same results in our earlier study^33^. (**D**) Whereas, TGs were significantly reduced in the leptin-treated group compared to their control. Collective data of two independent experiments are shown.

Leptin has an indispensable role in the weight loss related disorders like cachexia and anorexia nervosa, which has been associated with the low circulating plasma leptin levels^39^. To prove this, we measured the body weight upon leptin treatment of each mouse of both diet groups. With the diet-D (malnutrition diet), the body weights were significantly reduced in both control and infected groups compared to groups taken diet-A (normal diet). However, the leptin-treated diet-D infected group indicates a significant gain in comparison with control (Fig. 1A).

### Leptin has no effect on the blood glucose levels

Blood glucose is an important energy source for the survival and proper function of lymphocytes^40^. Hence, we measured the blood glucose levels the post-prandial blood glucose levels were found to be unaltered in both the diets of control and infected groups and could not found such an effect in the leptin-treated and untreated diet-D infected groups (Fig. 1B).

### Malnutrition and *L. donovani* infection affect serum leptin and Triglyceride (TGs) levels

Serum leptin is an optimal biomarker for malnutrition, and the serum TGs are best predictors of serum leptin^41^. Therefore, we measured the serum leptin and TGs in our test groups. In control groups, the serum leptin and TGs were significantly reduced in the diet-D compared to diet-A. Interestingly, we also observed a significant reduction in the serum leptin and TGs in the diet-A infected group compared to diet-A control group, which reinforces our hypothesis that leptin could play a protective role in VL pathogenesis and also indicates that TGs is a good predictor of circulating leptin. In subcutaneous leptin administration, we noticed a significant restoration of the serum leptin in the diet-D infected group compared to its untreated group (Fig. 1C). Furthermore, TGs were found to be significantly downregulated in the leptin-treated diet-D infected group (Fig. 1D).

### Leptin induces Th1 immune response hallmark IgG2a antibody

**Figure 2:**
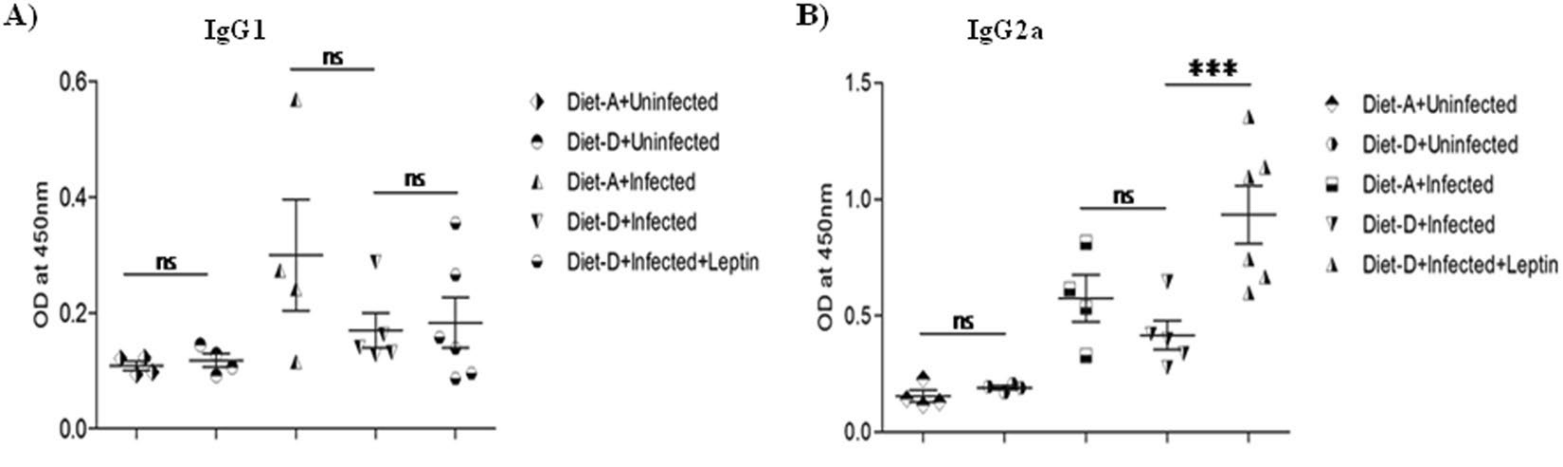
Serum IgG1 and IgG2a titers (OD at λ_450nm_). In comparison between uninfected groups and between infected groups of both the diets, the IgG1and IgG2a titers were found to be unaltered. Whereas in comparison between infected diet-D and its leptin-treated group, the IgG1 titers were found to be unaltered but the IgG2a titers were significantly induced in the leptin-treated group. Collective data of two independent experiments are shown.

The type of CD4+ Th-subset response determines the subclass and quantity of the IgGs, which is also influenced by the disease susceptibility or resistance^42^. Here, we measured the serum antibodies titers upon leptin treatment in each mice group of both diets. The serum IgG1 and IgG2a titers were found to be unaltered in both the diets of control groups and infected groups (Fig. 2A). Although the IgG1 titer was found to be unaltered, the IgG2a titer was significantly increased in the leptin-treated diet-D infected group compared to its untreated group (Fig. 2B), suggesting that leptin expand the host protective humoral immune response.

### Leptin controls *Leishmania* load in BALB/c mice visceral organs

**Figure 3:**
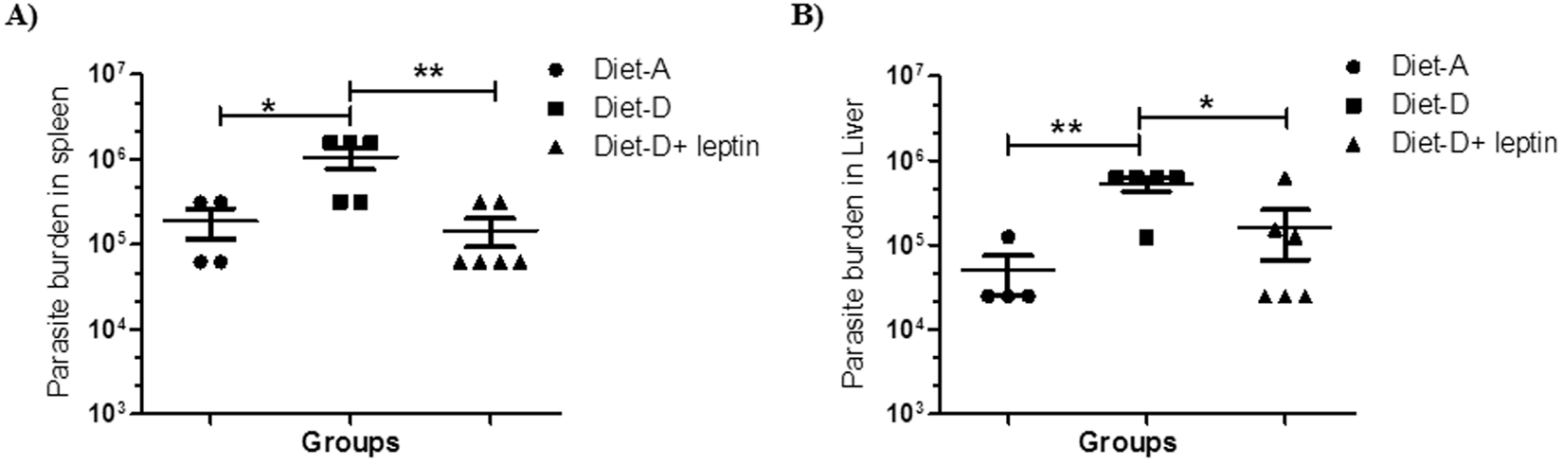
Parasite burden in Spleen and Liver of mice upon leptin treatment initiated simultaneously after infection. (**A**) The parasite load in the spleen of Diet-A, diet-D, and leptin treated diet-D groups (**B**) Parasite burden in the liver of diet-A, diet-D and leptin treated diet-D groups. In comparison between the diet-D infected and its leptin-treated group, the parasite load was significantly reduced in both the Spleen & Liver. Collective data of two independent experiments are shown.

The intravenous injection of metacyclic promastigotes could directly affect the visceral organs by infecting the tissue macrophages and dendritic cells (DCs), thereby spreads the infection by devastating the host immune response^43^. Thus, we analysed the parasite burden in infected mice group of both the diets and leptin treated diet-D infected groups. The parasite burden in the spleen and liver of the diet-D infected group were significantly higher as compared to diet-A infected group. Whereas, in the leptin-treated diet-D infected group, the parasite burden in the spleen and liver was significantly less as compared to its untreated group (Fig. 3A and B).

### Leptin induces hepatic granulomatous response to clear the infection

**Figure 4:**
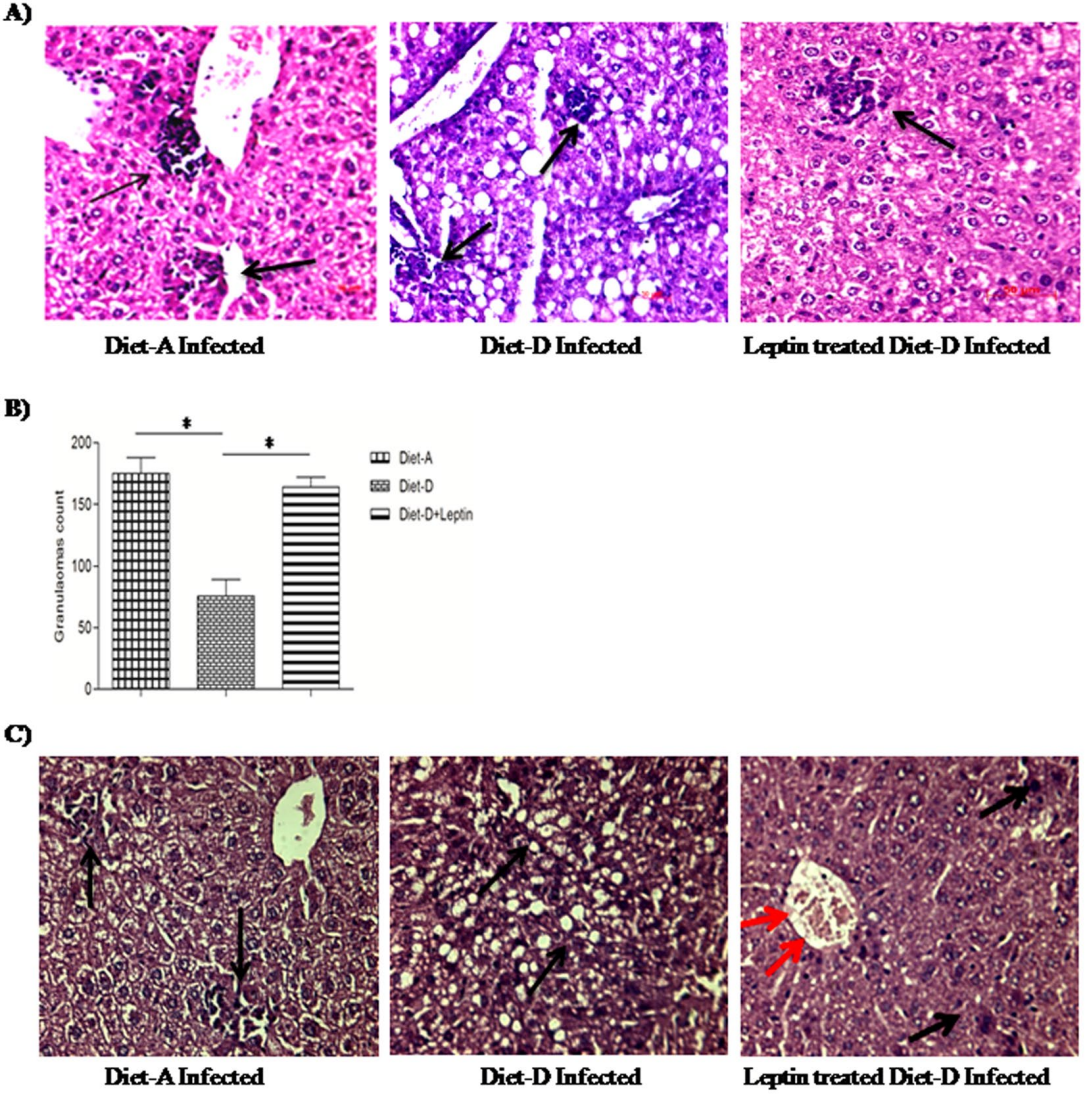
Arrow indicates the structural organization and number of granulomas in H&E stained liver sections (20X). (**A**) Diet-A infected group with large and well-organized granulomas, Diet-D infected group with tiny granulomas, and leptin-treated diet-D infected group with well-organized granulomas. (**B**) Granulomas count; Diet-A, Diet-D and leptin-treated diet-D group. (**C**) Hepatic tissue degenerative changes; In the diet-A, infected groups showed, mild hepatic degeneration at the centrilobular region and proliferation of fibrous tissue at the peribiliary region. In the diet-D infected groups showed moderate to severe vacuolar degeneration was noticed at the periportal and centrilobular region. In the leptin-treated diet-D infected, most of the hepatocytes appeared normal, portal and periportal region along with the bile duct appeared normal, and mild hepatic degeneration was noticed in few places of the centrilobular region. Collective data of two independent experiments are shown.

Rapid granuloma formation accelerates the parasite killing in the liver that can be facilitated by IFN-γ+ T-cells and NO producing macrophages in response to kuffer cells produced chemokines, and myeloid DCs derived IL-12^44^. Histological studies on the hepatic tissue confirmed that the size, integrity, and the number of the granulomas were significantly reduced in the diet-D (75.5 ± 13.5) compared to diet-A (175 ± 13). However, the leptin-treated diet-D infected group showed a significant increase in the size and number (164 ± 8; p ≤ 0.05) of granulomas compared to its untreated group (Fig. 4A and B).

The wound healing and angiogenesis activities of leptin were also observed to moderate severe vacuolar degeneration observed in the diet-D infected group. Vacuolar degeneration was significantly healed in the leptin-treated diet-D infected group, whereas the mild hepatic degeneration were curbed to the few places of the centrilobular regions. Interestingly, we also noticed a mild hepatic degeneration at the centrilobular region and proliferation of fibrous tissue at the peribiliary region in the diet-A infected group (Fig. 4C). It suggests that the *L. donovani* infection itself cause hepatic degeneration. However, control groups on both the diets displayed normal hepatocytes, portal, and periportal regions along with in all regions of the liver sections (data not shown).

### Leptin induces Th1 polarization and diminishes Th2 response in the spleen

**Figure 5:**
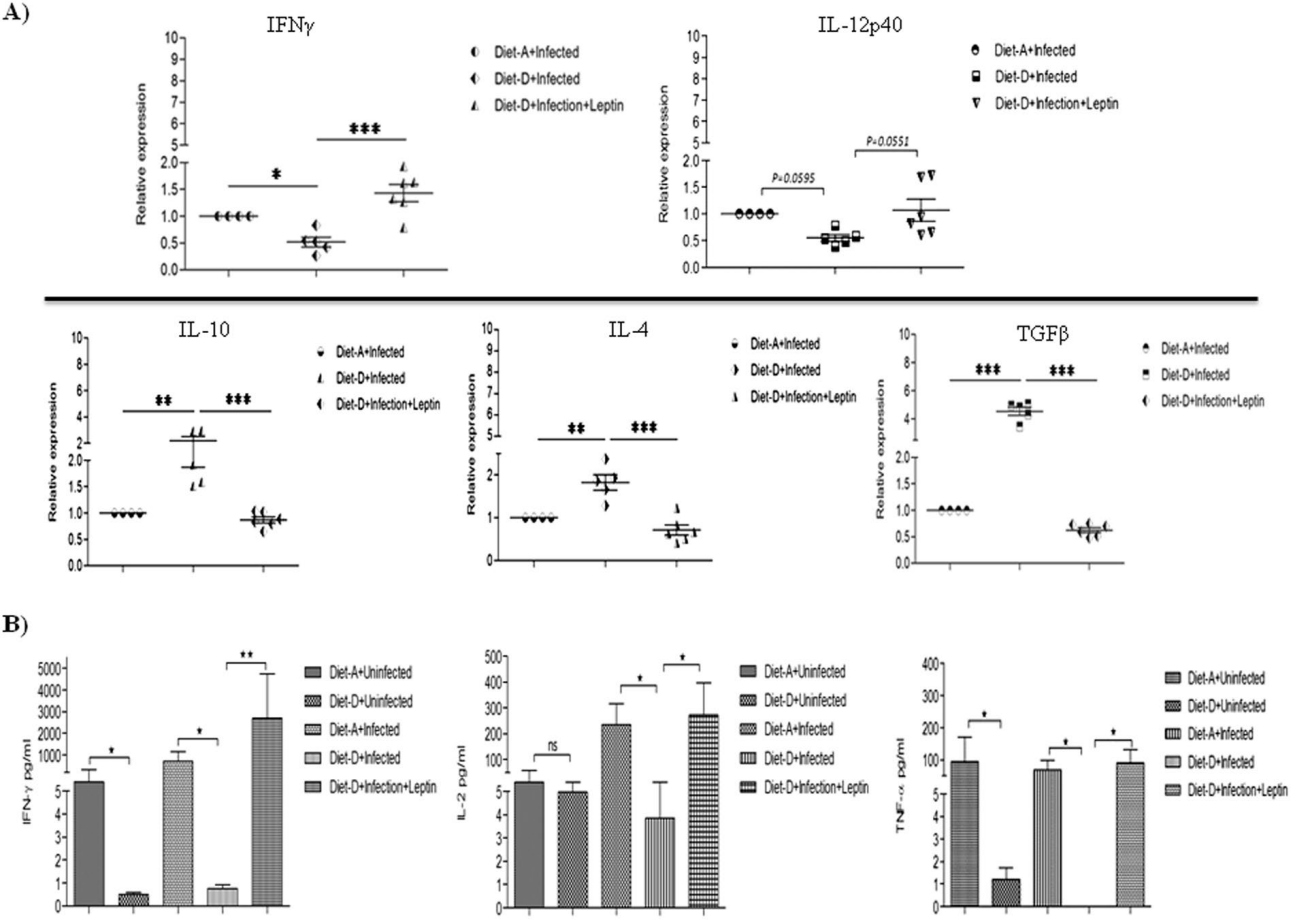
Gene expression analysis in the spleen of mice. (**A**) RT-qPCR analysis of Th1 and Th2 specific cytokines relative expression. In comparison between the infected groups of both the diets, the Th1 cytokines such as IL-12p40 (p = 0.0595) and IFN-γ was significantly downregulated, and the Th2 cytokines such as IL-10, IL-4, and TGF-β were significantly upregulated in the diet-D. Whereas, in the comparison between the infected diet-D and its leptin-treated group, IL-12p40 (p = 0.0551) and IFN-γ were significantly upregulated, and the Th2 cytokines were significantly down-regulated in the leptin-treated group. (**B**) Flow cytometry analysis of Th1 specific cytokine concentration (pg/ml) in SLA-stimulated splenocytes culture supernatant with mean ± SD. In comparison between the uninfected groups of both the diets, IFN-γ and TNF-α were produced at the lower rate in the diet-D compared to diet-A. In comparison between the infected groups of both the diets, IFN-γ and IL-2 were produced at the lower rate in the diet-D compared to diet-A. Whereas, in the comparison between the infected diet-D and its leptin treated group, IFN-γ, IL-2, and TNF-α were produced in abundant quantity in the leptin-treated group. Collective data of two independent experiments are shown.

Typically, the pathogenesis of VL is associated with elevated *Leishmania*-specific Th2 response and decreased APCs, leads to inhibition of protective T-cell response^45^. Hence, we measured the Th1/Th2 specific cytokines in each mice group of both the diets and leptin treated diet-D infected groups. The RT-qPCR results showed that in infected groups, the relative expression of proinflammatory cytokines such as IL-12p40 and IFN-γ were significantly downregulated in the diet-D compared to diet-A. Whereas, the relative expression of anti-inflammatory cytokines such as IL-10, IL-4, and TGF-β were significantly upregulated in the diet-D compared to diet-A. Similarly results were also observed in control groups of both diets (Supplementary Figure 2). The leptin-treated diet-D infected group showed a significant upregulation of IFN-γ, IL-12p40 expression and significant down-regulation of IL-10, IL-4, and TGF-β cytokines compared to its untreated group (Fig. 5A).

The immune dysfunction during active VL caused by *L. donovani* has been associated with lymphocytes incapability to produce cytokines in response to *Leishmania* specific antigen stimulation^46^. Flow cytometry results show that IFN-γ and tumor necrosis factor TNF-α cytokines production in the splenocytes culture supernatant were drastically reduced upon SLA-stimulation in diet-D compared to diet-A in both groups. The IL-2 secretion, on the other hand, was found to be unaltered between the control groups of both the diets, but was diminished in the diet-D infected group compared to diet-A. Leptin-treatment restored the IFN-γ, IL-2, and TNF-α cytokines in the diet-D infected group (Fig. 5B).

### Leptin induces CD8+ T-cells infiltration into the spleen

**Figure 6:**
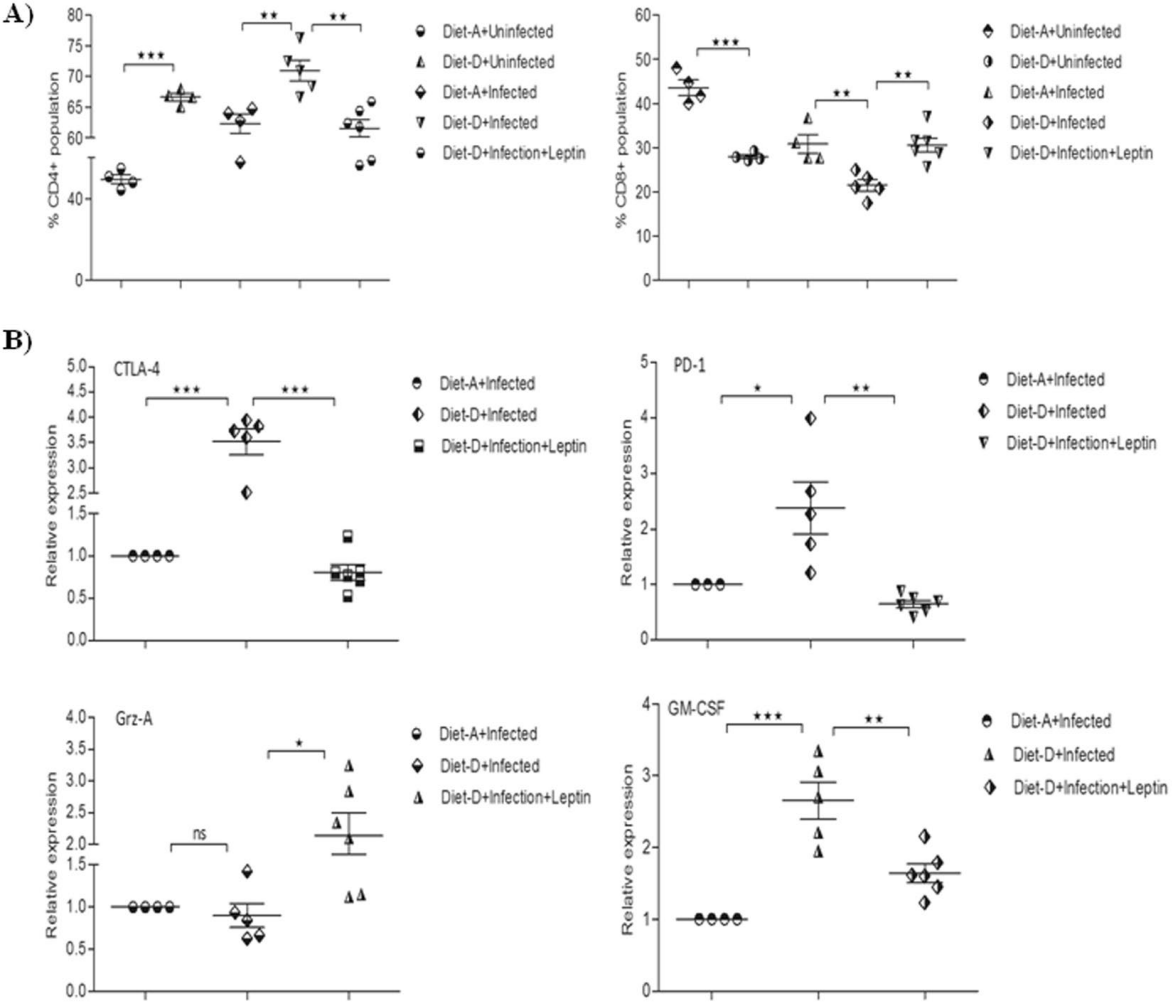
Percentage of T-cell subpopulations in VL spleen of mice. (**A**) In comparison between the uninfected groups and between the infected groups of both the diets, the CD4+ population was significantly increased and the CD8+ population was significantly decreased in the diet-D. Whereas, in the comparison between the infected diet-D and its leptin-treated group, the CD8+ population was significantly increased and the CD4+ population was significantly decreased. (**B**) RT-qPCR analysis of exhaustive T-cell markers, Grz-A, and GM-CSF relative expression. In comparison between the infected groups of both the diets, the exhaustive T-cell markers (CTLA-4 and PD-1) and GM-CSF were significantly upregulated, and Grz-A was found to be unaltered in the diet-D. Whereas, in the comparison between the infected diet-D and its leptin-treated group, both the exhaustive markers and GM-CSF were significantly downregulated, and Grz-A was significantly upregulated in the leptin-treated group. Collective data of two independent experiments are shown.

Although a very little is known about the actual role CD8+ T-cells in VL, recent studies has shown CD8+ T cells have an anergic or exhausted phenotype, as indicated by high expression of CTLA-4, PD-1, and IL-10, which may affect the protective capacity of these cells during clinical VL^47^. In infected mice, the percentage of CD4+ T-cells were significantly increased over the CD8+ T-cell population in the diet-D compared to diet-A. However, in the leptin-treated diet-D infected mice the above scenario was reversed significantly (Fig. 6A). Hence, we can associate the increased CD8+ T cells population in the leptin-treated mice might with suppression of parasite dissemination in VL mice.

### Leptin downregulates exhaustive T-cell markers, GM-CSF and upregulates Grz-A mRNAs expression in *Leishmania* infected BALB/c mice spleen

Upregulation of T-cell exhaustion markers indicates the favourable environment for high pathogenicity due to the unresponsiveness of T-cells to invading pathogen. During chronic VL, the CD4+ T-cell anergy is apparent due to the abundant expression of CTLA-4, PD-1/B7.H1 that leads to the massive production of TGF-β, which suppressed normal macrophage activity that leads persistence of infection^48,49^. Therefore, we analyzed the expression of these exhaustive T cells markers in each mice given the different diets and leptin treatment. In the infected groups, the relative expression of exhaustive T-cell markers such as CTLA-4 and PD-1 along with GM-CSF was significantly upregulated, and Grz-A was unaltered in the diet-D compared to diet-A. Although the leptin-treated diet-D infected group, the relative expression of Grz-A was significantly upregulated, CTLA-4, PD-1, and GM-CSF were significantly downregulated compared to its untreated group (Fig. 6B). Similarly, a comparison between the control groups of both diets was shown in (Supplementary Figure 3). In our study, the GM-CSF expression was 5-folds higher in the diet-A infected group compared to its control group (data not shown), which substantiates the early report on the hepatic GM-CSF mRNA expression^50^. The reduced GM-CSF expression with the leptin treatment in malnutrition coupled VL might be an indicator of reduced parasitized favoured monocytes proliferation, which controls parasite dissemination in the spleen.

### Leptin reduces arginase activity in the BMφ

**Figure 7:**
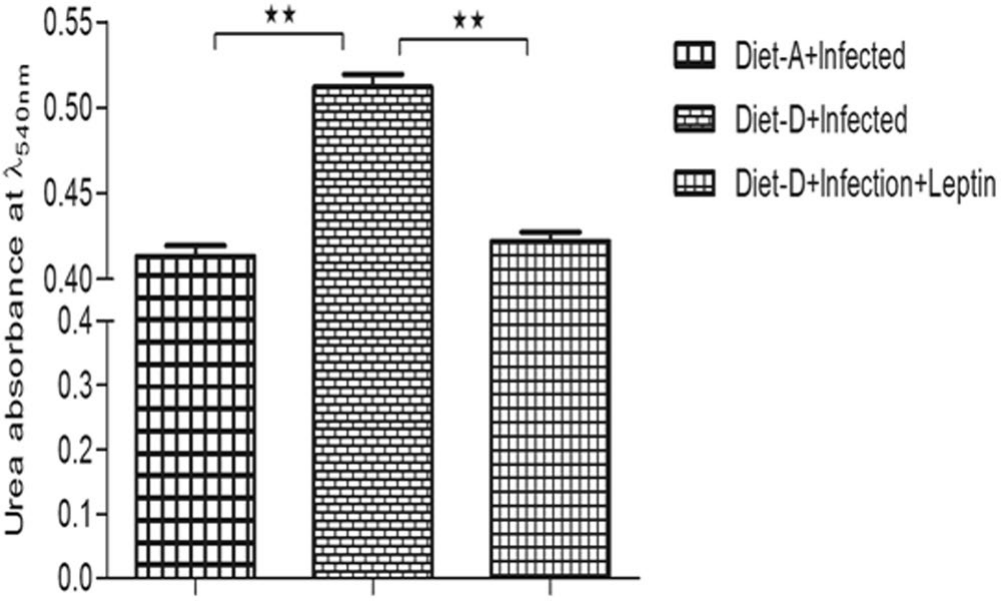
An absorbance of end product urea with mean OD (λ_540nm_) ± SD during catabolism of L-arginine by arginase activity of BMφ. In comparison between the infected groups of both the diets, the absorbance of urea (i.e. arginase activity) is significantly higher in the diet-D. On the other hand, in the comparison between the infected diet-D and its leptin-treated group, it was significantly downregulated in the leptin-treated group. Collective data of two independent experiments are shown.

*L. donovani* infection usually evades the inducible nitric oxide synthase (iNOS)-dependent killing mechanism by macrophages through elevating arginase expression^51^ (Biswas *et al*., 2011). Arginase mediated catabolism of L-arginine leads to the production urea at the end of the reaction as by forming intermediates like ornithine, it further catabolized to the polyamines, which are essential components of host cell division and parasite survival. The increased arginase activity in malnutrition, as well as several pathological conditions, depletes the L-arginine at microenvironment, which affects the monocytes dependent immune responses^52,53^. Concurrently, we analyzed arginase dependent macrophage activation in an infected group of both diets and leptin treated diet-D infected groups. In the infected groups, the arginase enzyme activity was significantly increased in the diet-D compared to diet-A. Whereas, in the leptin-treated diet-D infected group, the arginase activity was significantly reduced compared to its untreated group (Fig. 7), suggesting the macrophages dependent killing of the intracellular parasite upon leptin treatment.

## Discussion

VL is delineated by the inability to control *L. donovani* infection associated with an intense T-cell insensitivity to *Leishmania* antigens and production of IL-10 cytokine^54–56^. The crucial role of the host specific cell-mediated immunity is illustrated by the increased risk of developing clinical illness in case of malnutrition or concomitant immunosuppressive diseases, such as HIV co-infection^3^. The nutritional deficiency impairs adaptive and innate immunity that is essential for the defence against infection *Leishmani5* ^57,58^. VL is prevalent in poor people invariably suffering from malnutrition that could be endorsed with reduced circulating leptin levels. However, the leptin level during active VL infection in human has not been demonstrated yet.

Leptin has been identified as a hallmark biomarker in malnutrition condition^41^. Concurrently, we also observed a drastic fall in the serum leptin level in infected mice fed with normal diet confirms our previous hypothesis that the circulating leptin levels might downregulate during *Leishmania* infection^33,37^. The low circulating leptin has been shown to strongly correlate with reduced TGs in tuberculosis^59^ and to perturb the host lipid profile in leishmaniasis^60^, which in turn could plausibly affect the circulating plasma leptin. Subsequently, infection induced low plasma leptin could facilitate an impaired T-cell response as reported in tuberculosis^59^. In spite of the drastic collapse in the serum leptin levels observed following *L. donovani* infection in both the diets, the disease severity was predominant in the diet-D compared to diet-A, highlighting the importance of other nutritional factors during *L. donovani* infection. Probably, vitamin-E^61^ or zinc^62^ could restore the leptin production in these conditions.

Splenic infection of the diet-D fed animals might have accelerated the maturation of double positive T-cells inclined towards CD4+^63^ rather than CD8+ and could explain increased CD4+/CD8+ T cells ratio (Supplementary Figure 4), something also found to be correlated with pulmonary tuberculosis in leptin-deficient mice^26^. Hence, we speculated that the increased CD4+ population in diet-D could be exhausted CD4+ of Th2 phenotype in considering discernible upregulation of IL-10, IL-4, and TGF-β mRNA expression. Simultaneously, a massive expression of CTLA-4 and PD-1 in the diet-D merely justifies that the effector CD4+^48^ and CD8+ T-cells are in an anergic state during chronic VL^47,64,65^. Despite the indistinctive role of CD8+ T-cell in human VL^47^, the blockade of these anergic markers during *L. donovani* infection results in increased survival of CD4+ and CD8+ T cells function and dramatically increased reactive oxygen species production in co-cultured monocyte-derived phagocytes to clear intracellular parasites^49,66,67^ and the production of IFN-γ followed by cure entails the protective role of CD8+ T-cells in VL^47^.

In above circumstances, exogenous recombinant leptin has retained the effective T-cell function and reduced splenic parasite burden compared with non-treated infected mice fed with normal diet. The decrease in parasite load correlated with an induction of innate immune response in antigen-presenting cells that showed an increase in nitric oxide, enhanced pro-inflammatory cytokines (IFNγ, IL-12, and IL1β) response in the splenocytes, indicating host-protecting Th1 response mediated by leptin^37^. Moreover, leptin-treated infected mice induced IFNγ production from both CD4+ and CD8+ T cells, compared with non-treated infected mice^37^ replicating earlier report, in respiratory and gastrointestinal infected malnourished children^19^. Increased splenic CD8+ T-cell number and Grz-A production entail the healing of disease^67^ arbitrated by leptin. Typically, the CD4+ T-cell milieu influences the B-cell differentiation and IgG sub class switching phenomenon. The overall CD4+ Th1 response relates to the IgG2a titers and Th2 response overlaps with IgG1titers^68,69^. In our study, the IgG2a antibody titer was raised up with leptin treatment, reinforcing the stimulation of protective humoral immunity.

Granulomatous response is a crucial event to abrogate the parasite dissemination in the liver, which is entangled by the infiltration of T-cells^70–72^, activated macrophages, and monocytes influenced by Th1 cytokine environment^73–75^, to the site of infection. However, the indistinctive granulomatous response in malnutrition accounts for the impaired coordination between innate and adaptive immune cells in response to the *L. donovani* infection. As shown in previous report with 4% protein diet^63^, a moderate to severe vacuolar degeneration at the periportal and centrilobular regions of the hepatic tissue indicates an exacerbated necrosis-like death of the cells^76^ or tissue atrophy^77^ in malnutrition upon *L. donovani* infection. The exogenous leptin treatment has moderately restored the granulomatous response in pulmonary tuberculosis of leptin-deficient mice^31^ and also substantially prevented the tissue degeneration^78^ and angiogenesis activities^79,80^.

In murine infection with arginase-deficient *L. major*, it was demonstrated that impaired priming of T cells can result in PD-1 overexpression, impairment of acquired immunity, and exhaustion^51^. However, *L. donovani* infection usually evades the inducible nitric oxide synthase (iNOS)-dependent macrophages activation through elevating arginase expression, which is identical to the IL-10 function^13,76^. In our study, an increased arginase activity in the BMφ of diet-D certifies a higher disease severity as enrolled in the HIV co-infected visceral leishmaniasis^81^. The increased arginase activity in malnutrition coupled VL depletes the L-arginine at microenvironment, which impairs macrophage dependent T-cell activation and proliferation^82,83^. In our study, we observed the same leptin therapy has shown to reduce the arginase activity in diet-D infected mice, suggesting its direct role in the T-cell activation and functions. Moreover, elevated GM-CSF expression in the spleen indicates the rapid dissemination of infection via the induced proliferation of non-microbicidal monocytes exploited by the parasites for purine nucleotide machinery, which is absent in the *Leishmania* ^84^. Hence, we speculated that the *Leishmania* exploited monocytes or macrophages might be immature or alternatively activated with the lack of anti-microbial activity. Moreover, immature myeloid cells (CDb11 + Ly6C+) has been shown to rapidly expand in the bacterial (*Listeria monocytogenes*), fungi (*Candida albicans*), and parasitic (*Trypanosoma cruzi* and *L. major*) infections^85–87^, suppressing the pathogen-specific CD4+ and CD8+ T-cell responses^88,89^. Additionally, alternative macrophages could emerge into the Th2 environment that facilitates the parasite dissemination in the host^90^ supporting our speculation regarding the fate of monocytes and macrophages in *L. donovani* infection. However, leptin could have regulated the aforementioned parasite favorable events, in such a way that controlled the splenic infection.

In conclusion, we provide evidence for that exogenous leptin can restored the suppression of anergic T cells and favour GM-CSF expression in monocytes in malnutrition coupled *L. donovani* infection in BALB/c mice. Furthermore, since *L. donovani* infection lowered circulating leptin levels, leptin treatment could a way to overcome immunosuppression during VL. However, further studies are needed to investigate the fate of leptin in the clinical settings to credibly establish the immunotherapeutic role of leptin during visceral infection.

## Methods

### Parasite culture

Promastigotes strain (DD8) of the *L. donovani* was obtained from ATCC (American type culture collection, U.S.A.) and cultured in Medium-199 (Sigma) supplemented with 15% heat-inactivated fetal bovine serum (FBS), 20 mM HEPES, pH7.4, 4 mM NaHCO_3_, 100 U/ml of penicillin and 100 mg/ml of streptomycin (Sigma) at 25 °C ± 1. The *L. donovani* parasites used for infection were passaged in susceptible golden Syrian hamster as describe previously^91^.

### Animals and Experimental diets

In this study, 8-9 weeks old female BALB/c mice (average body weight 28–32 g) were used for experiments. Experimental diets were obtained from National Centre for Laboratory Animal Sciences (NCLAS, Hyderabad). We have used two different diets for this study such as diet-A and diet-D. The diet-A (normal diet) consists of 21% of protein and sufficient zinc and iron and diet-D (malnutrition diet) consists of 1.25% of protein, deficient in zinc and iron. Usually, the zinc deficiency implies the protein-energy malnutrition^92^ however, the iron deficiency leads mainly to anaemia and which is more prevalent in endemic areas of developing countries.

### Ethical license and Experimental infections

The animal experiment for this study was approved by the Institutional animal ethical committee, University of Hyderabad, Hyderabad (UH/IAEC/2014/RM/14). In addition to this all methods were performed in accordance with the relevant guidelines and regulations. BALB/c mice (n = 23) were divided into 5 groups, of which, uninfected (control) groups of each diet (n = 4), infected diet-A (n = 4), infected diet-D (n = 5), and infected diet-D supplemented with the leptin (n = 6). Each group was fed for 6-weeks with an average of 3.8 g/mouse/day of the respective diet.

### Pre-assessment of malnutrition

In a different set of experiment, it was pre-assessed for the malnutrition state after 3-weeks of feed. In order to this, we measured the serum leptin followed by other relevant parameters like body weight, serum triglycerides (TGs). Hence, the experimental animals (n = 8) were divided into two groups (n = 4) based on their diet as shown in Fig. 1 (Supplementary Figure 1).

### Mice infection and leptin treatment

Mice were infected with freshly derived metacyclic promastigotes (5 × 10^6^) derived from amastigotes that were isolated from infected mice and purified by Ficoll density gradient method. Simultaneously, leptin (5 μg/day) was administrated to the diet-D infected group through subcutaneous route and monitored for next 3-weeks. After completion of total 6-weeks, the body weight of each mice group was recorded, and then post-prandial blood glucose concentration was monitored using ACCU-CHEK^®^ kit by tail vein puncture. Then mice were euthanized; blood was withdrawn through the retro-orbital puncture and the serum was separated. The visceral organs (i.e. spleen and liver), and bone marrow were collected for further experiment.

### Quantitative estimation of the serum leptin and Triglycerides

Serum leptin was estimated by using Mouse Leptin enzyme-linked immune sorbent assay (Sigma). Serum TGs were quantified by an enzymatic method using Prism Diagnostics kit protocol. The color intensity was measured at λ_505nm_ using spectrophotometer (HITACHI U-2910).

### Quantitative estimation of the serum IgG1 and IgG2a

Serum IgGs were quantified by standard indirect ELISA method^93^. The color intensity was measured at λ_450nm_ using microplate reader (TECAN).

### Estimation of *Leishmania* burden in the spleen and liver

Parasite burden in BALB/c mice spleen and liver samples were determined by limiting dilution assay method as described previously^94^.

### Histopathological studies on hepatic tissue

Liver tissue sections were collected from all mice groups and fixed in 10% formalin saline buffer and stained with haematoxylin-eosin (H-E) (CDFD, Hyderabad). Inflammatory foci/granuloma formation and degenerative/necrosis changes were examined under the light microscope (Zeiss Axioplan 2 imaging microscope). The numbers of granulomas per fifty fields were counted at 20x using a light microscope (Leica).

### Gene expression analysis of cytokines in the spleen

For the gene expression analysis, real-time quantitative (RT-q) PCR was used. Briefly, total RNA was isolated from the spleen tissue using Nucleospin RNA kit protocol. Of which, 500 ng of RNA was used as template for complementary DNA (cDNA) synthesis using Takara PrimeScript™ cDNA synthesis kit. Gene-specific primers for IFN-γ, IL-12p40, IL-10, IL-4, TGF-β, Granzyme (Grz)-A, cytotoxic T-lymphocyte antigen (CTLA)-4, PD-1, and granulocyte macrophage-colony stimulating factor (GM-CSF) were designed using Primer Express Software (Table 1). The cDNA was amplified by using SYBR® Premix Ex Taq™ (Takara) in an ABI Prism 7300 Sequence Detector (Applied Biosystems) which was programmed for 2 min at 50 °C, 10 min at 95 °C, 15 sec at 95 °C (40 cycles) and 1 min at 60 °C. The relative expression of genes as mentioned above was calculated using StepOnePlus™ software based on their threshold cycle (Ct) values in respect to glyceraldehyde 3-phosphate dehydrogenase (GAPDH) Ct values.

**Table 1:** Gene-specific primers were used to amplify the target mRNA of cytokines and T-cell makers by RT-qPCR.

S. No	Primer name	Sequence (5′—>3′)
1	GM-CSF	FP: 5′-GCCATCAAAGAAGCCCTGAA-3′RP: 5′-GCGGGTCTGCACACATGTTA-3′
2	IL-10	Forward 5′-GGTTGCCAAGCCTTATCGGA-3′Reverse 5′-ACCTGCTCCACTGCCTTGCT-3′
3	IL-12p40	Forward 5′-GGAAGCACGGCAGCAGAATA-3′Reverse 5′-AACTTGAGGGAGAAGTAGGAATCG-3′
4	IFN-γ	Forward 5′-TCAAGTGGCATAGATGTGGAAGAA-3′Reverse 5′-TGGCTCTGCAGGATTTTCATG-3′
5	IL-4	Forward 5′-ACAGGAGAAGGGACGCCAT-3′Reverse 5′-GAAGCCCTACAGACGAGCTCA-3′
6	TGF-β	Forward 5′-TGACGTCACTGGAGTTGTACGG-3′Reverse 5′-GGTTCATGTCATGGATGGTGC-3′
7	GAPDH	Forward 5′-CAAGGCTGTGGGCAAGGTCA-3′Reverse 5′-AGGTGGAAGAGTGGGAGTTGCTG-3′
8	Granzyme-A	FP: 5′-CAT TGG AGG AGA CAC GGT TGT TCC-3′RP: 5′-CTC TTT CCC ACG TTA CAG TGG GC-3
9	CTLA-4	FP: 5′-GGACTTGGCCTTTTGTAGCCCT-3′RP: 5′-ATT CAC ATG GAA AGC TGG CGA CAC-3′
10	PD-1	FP: 5′-CCTGGTCATTCACTTGGGCTGTG-3′RP: 5′-GGT GGC ATT TGC TCC CTC TGA-3′

### Quantitative estimation of cytokines in SLA-stimulated splenocytes culture supernatant

Flow cytometry experiments were performed to quantify the splenocyte culture supernatant cytokines using the cytokine bead array (CBA) kit. Briefly, splenocytes (5 × 10^6^/ml) were seeded into 12-well culture plate in complete RPMI-1640 medium and cells were stimulated with SLA (60 μg) for 72 h at 37 °C in CO_2_ incubator. For the cytokines analysis, splenocytes culture supernatant was processed according to BD™ CBA kit instructions and analyzed by using BD LSRFortessa™ and FCAP Array™ v3.0 software.

### Cell surface staining for flow cytometric analysis of T-cell subpopulation

Splenocytes were stained with fluorescently labeled cell-surface markers such as Fluorescein isothiocyanate (FITC)-CD3, Phycoerythrin (PE)-CD4 and Allophycocyanin (APC)-CD8 (eBiosciences) to analyze T-cells and its subpopulation. Briefly, splenocytes (5 × 10^6^/ml) were washed with ice-cold PBS and resuspended in 100 μl PBS containing 1 μg FITC-CD3, 0.5 μg CD4-PE, and 0.5 μg CD8-APC, and incubated for 30 min on ice in the dark. Finally, cells were resuspended in FACS staining buffer and analyzed by flow cytometry (BD LSRFortessa™, FACSDiva™ software).

### Quantitative estimation of arginase activity

Arginase activity was measured in the BMφ cell lysates. Briefly, bone marrow cells from all experimental groups were cultured by stimulating with 25 ng/ml macrophage colony-stimulating factor (M-CSF) for 7-days at 37 °C in 5% CO_2_. Of which, BMφ (5 × 10^5^/ml) were seeded into the 24-well plate and stimulated with SLA (60 μg). The culture plate was incubated at 37 °C in 5% CO_2_ for 72 h. Next, cell lysates were prepared by using 100 μl radio immuno-precipitation assay (RIPA) lysis buffer containing 1% proteinase inhibitor cocktail. 100 μl of cell lysates (25 μg) were incubated with 10 μl MnCl_2_ (10 mM) at 56 °C for 10 min to activate the arginase enzyme. The hydrolysis of 100 μl L-arginine (0.5 M, pH-9.7) was performed by incubating with activated lysates at 37 °C for 20 min. The reaction was terminated with 900 μl of H_2_SO_4_ (96%)/H_3_PO_4_ (85%)/H_2_O (1v/3v/7v) followed by incubation with 40 μl of 9% α-iso nitroso propiophenone (prepared in 100% ethanol) at 95 °C for 30 min. The hydrolysis of L-arginine by arginase results in the production of urea at the end, whose color intensity was measured at λ_540nm_ using spectrophotometer (HITACHI U-2910) to address the arginase activity in BMφ.

### Statistical analysis

Data analysis was performed using one-way ANOVA, and significance was calculated by Newman-Keuls multiple comparison tests and results are expressed as standard errors mean (SEM). Few experimental results are expressed as the mean ± standard deviation (SD) using unpaired t-test (GraphPad Prism 5). Statistical significance was considered as p ≤ 0.05, p ≤ 0.01, and p ≤ 0.001 and represented by *, **, and *** respectively.

## Electronic supplementary material

Supplementary information
