# Supplementary material for: Leptin regulates Granzyme-A, PD-1 and CTLA-4 expression in T cell to control visceral leishmaniasis in BALB/c Mice
Source: Sci Rep. 2017 Nov 7;7:14664. doi: 10.1038/s41598-017-15288-7 (PMC5676676; doi:10.1038/s41598-017-15288-7)
Supplement: Supplementary file 1 — Supplementary information [file 41598_2017_15288_MOESM1_ESM.pdf]

Supplementary Information

Leptin regulates Granzyme-A, PD-1 and CTLA-4 expression in T cell to control visceral leishmaniasis in BALB/c Mice

Alti Dayakar[1], Sambamurthy Chandrasekaran[1], Jalaja Veronica[1], Vadloori Bharadwaja[1], Radheshyam Maurya[1*]

Malnutrition status is approved with the diet-D based on the reduction in serum leptin, TGs and body weight

We have noticed a significant reduction in the body weight ($p \leq 0.01$), serum leptin ($p \leq 0.05$), and serum TGs ($p \leq 0.01$) in the diet-D group compared to diet-A, after 3-weeks of initial feed (Fig.1). Based on this, we have approved the malnutrition status of our experimental animals. In our study, the loss of body weight was about 30%, which represents the moderate malnutrition state as shown by the previous report (Cuervo-Escobar et al., 2014). In addition, blood glucose and serum cholesterol levels were not significantly varied between both the diet groups (data not shown).

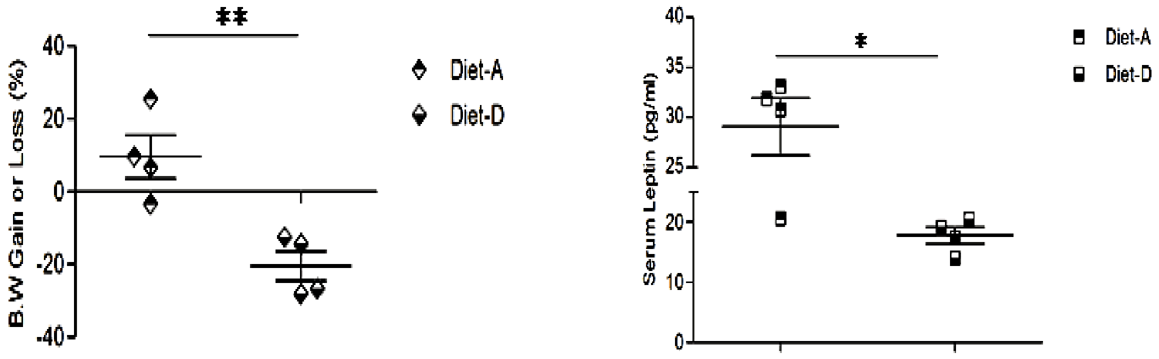

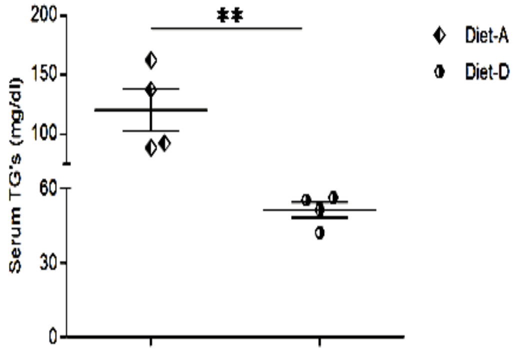

Figure 1: Pre-assessment of malnutrition status after 3-weeks of initial feeding. Body weight, serum leptin, and serum TGs were significantly reduced in the diet-D compared to diet-A. Analysis of variance was done using one-way ANOVA, significance was calculated by Newman-Keuls multiple comparison test and $p \leq 0.05$ and $p \leq 0.01$ represents * and **.

Malnutrition induces Th2 polarisation and affects Th1 cytokines expression

Malnutrition itself can be enumerated for default immune setting, characterized by Th2 polarization and Th1 decline. Herewith, in comparison between the uninfected groups of the diets, the relative expression of IL-12p40 ($p \leq 0.05$) was significantly downregulated and IFN-γ was unaltered in the diet-D compared to diet-A. On the other hand, the relative expression of IL-10 (p=0.09), IL-4 ($p \leq 0.05$), and TGF-β (p=0.076) were upregulated in the diet-D compared to diet-A (Fig 2).

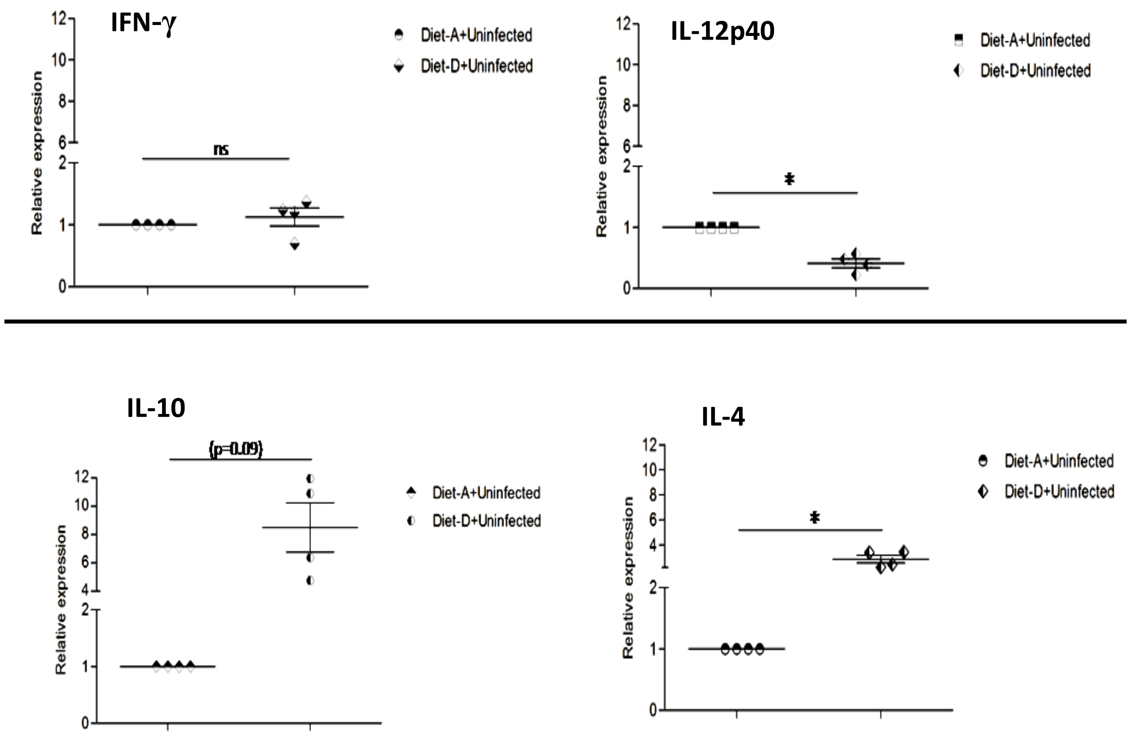

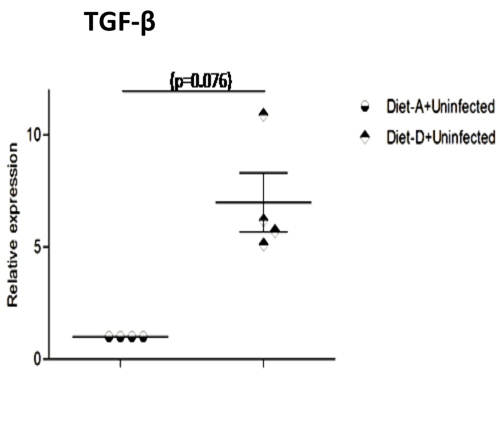

Figure 2: RT-qPCR analysis of Th1 (panel-A) and Th2 (panel-B) cytokines relative expression with SEM. In comparison between the uninfected groups of both the diets, the IFN-γ was unaltered and IL-12p40 was significantly downregulated, and the IL-10 (p=0.09), IL-4, and TGF-β (p=0.076) were upregulated in the diet-D. Significance was indicated by *$p \leq 0.05$.

Malnutrition upregulates splenic exhaustive T-cell markers and GM-CSF expression

Malnutrition tends to T-cell unresponsiveness at microenvironment and immature monocytes infiltration into the tissues from BM via blood circulation. Here, the T-cell unresponsiveness can be authenticated by the abundant expression of exhaustive markers and the immaturity of monocytes plausibly addressed by a massive expression of GM-CSF. In comparison between the uninfected groups of both the diets, the relative mRNA expression of CTLA-4 and PD-1, and GM-CSF was significantly ($p \leq 0.01$) upregulated and Grz-A, a potent anti-microbial component of cytotoxic T-lymphocytes, was found to be unaltered in the diet-D compared to diet-A (Fig 3).

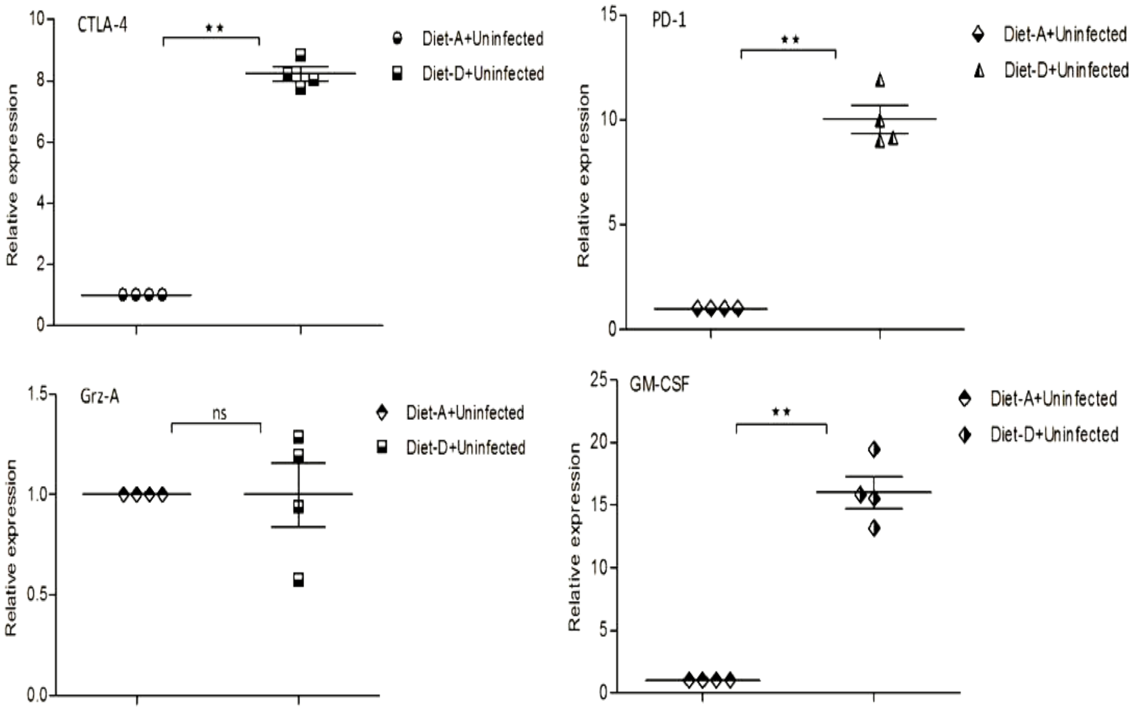

Figure 3: RT-qPCR analysis of CTLA-4, PD-1, Grz-A, and GM-CSF with SEM. In comparison between the uninfected groups of both the diets, exhaustive T-cell markers such as CTLA-4 and PD-1, and GM-CSF were significantly upregulated and Grz-A was found to be unaltered in the diet-D. Significance was indicated by $^{**}p \leq 0.01$.

Malnutrition upregulates CD4+/CD8+ T cells ratio in spleen of VL Mice

Malnutrition itself dysregulates the CD4+/CD8+ T-cell ratios in diet-D uninfected mice compared to diet-A uninfected mice. In comparison between the infected mice groups of both the diets, the percentage of CD4+/CD8+ T-cell ratios were significantly increased in the diet-D compared to diet-A. However, in the leptin-treated diet-D infected mice the above scenario was reversed significantly compared to non-treated diet –D infected mice. Hence, the increased CD8+ T cells population in the leptin-treated mice might with suppression of parasite dissemination in VL mice.

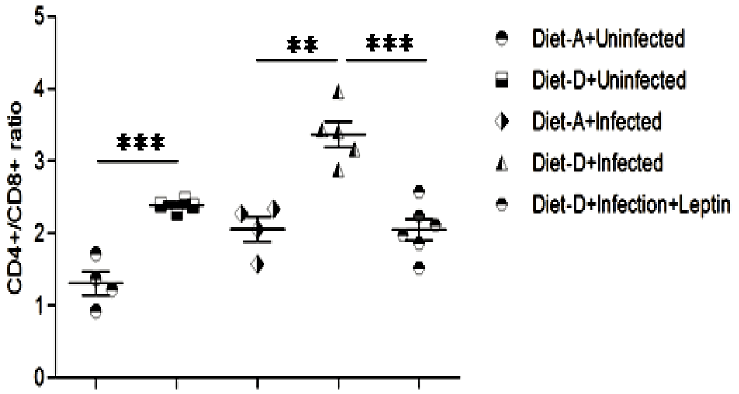

Figure 4: Percentage ratio of CD4+/CD8+ T cells in VL spleen of mice. In comparison between the uninfected groups and between the infected groups of both the diets, the CD4+/CD8+ population was significantly increased in the diet-D group. Whereas, the CD4+/CD8+ T cells ratio was significantly decreased in the leptin-treated diet-D infected group compared to the non-treated diet-D infected group. Significance was indicated by $^{**}p \leq 0.01$.
